# The role of microglia and the TLR4 pathway in neuronal apoptosis and vasospasm after subarachnoid hemorrhage

**DOI:** 10.1186/1742-2094-10-83

**Published:** 2013-07-13

**Authors:** Khalid A Hanafy

**Affiliations:** 1 Division of NeuroCritical Care, Department of Neurology, Harvard Medical School, Beth Israel Deaconess Medical Center, The Center for Life Science, 3 Blackfan Circle, Boston, MA 02215, USA

**Keywords:** TLR, TRIF, MyD88, SAH, Microglia

## Abstract

**Background:**

Although microglia and the Toll-like receptor (TLR) pathway have long been thought to play a role in the pathogenesis of aneurysmal subarachnoid hemorrhage (aSAH), thus far only correlations have been made. In this study, we attempted to solidify the relationship between microglia and the TLR pathway using depletion and genetic knockouts, respectively.

**Methods:**

Subarachnoid hemorrhage was induced in TLR4−/−, TRIF−/−, MyD88−/− and wild type C57BL/6 mice by injecting 60 μl of autologous blood near the mesencephalon; animals were euthanized 1 to 15 days after SAH for immunohistochemical analysis to detect microglia or apoptotic cells. Lastly, microglial depletion was performed by intracerebroventricular injection of clodronate liposomes.

**Results:**

On post operative day (POD) 7 (early phase SAH), neuronal apoptosis was largely TLR4-MyD88-dependent and microglial-dependent. By POD 15 (late phase SAH), neuronal apoptosis was characterized by TLR4- toll receptor associated activator of interferon (TRIF)-dependence and microglial-independence. Similarly, vasospasm was also characterized by an early and late phase with MyD88 and TRIF dependence, respectively. Lastly, microglia seem to be both necessary and sufficient to cause vasospasm in both the early and late phases of SAH in our model.

**Conclusion:**

Our results suggest that SAH pathology could have different phases. These results could explain why therapies tailored to aSAH patients have failed for the most part. Perhaps a novel strategy utilizing immunotherapies that target Toll like receptor signaling and microglia at different points in the patient’s hospital course could improve outcomes.

## Introduction

Aneurysmal subarachnoid hemorrhage (aSAH) is a devastating disease that affects 30,000 Americans each year [1,2]. A total of 30 to 40% of these patients will have delayed cerebral ischemia (DCI) from vasospasm, anywhere from 4 to 14 days after ictus, resulting in increased morbidity and mortality [3]. Few studies have addressed the molecular mechanisms that lead to DCI. The inciting event is the release of oxyhemoglobin from red blood cell lysis in the subarachnoid space [4,5]. The heme that is released from hemoglobin results in a significant cerebral inflammatory response [6]. To dissipate the heme burden, the resident cerebral macrophage or microglia can engulf heme, as demonstrated in mouse intracerebral hemorrhage models, and degrade it via heme oxygenase [7,8]. Furthermore, in primary macrophage culture, heme has been shown to be a specific agonist for the Toll-like receptor 4 (TLR4) [9].

In both SAH patients and mouse models, TLR4 expression is up-regulated in the brain [10-12]. However, little is known about the signal transduction that occurs downstream of TLR4 to cause DCI or what cells mediate DCI. That is, after stimulation of TLR4, the *MyD88* (myeloid differentiation primary response gene) pathway can facilitate further action by phosphorylation and activation of IRAK4 (IL-1 receptor associated kinase 4), which results in NF-kB-dependent inflammation [13]. Alternatively, TLR4 signaling can be transduced with the TRIF (toll receptor associated activator of interferon) pathway [13]. TRIF signaling results in a delayed NF-kB activation, similar to MyD88, and facilitates apoptosis [14].

The understanding of the immune cells involved in DCI is in its infancy. Neutrophils may play a role in DCI as systemic depletion resulted in improved cognitive performance and decreased vasospasm; however, their role in cerebral inflammation after SAH is likely indirect [15]. Neutrophils are not endogenous to the brain, nor do they have a published role in aneurysm formation. Resident macrophages of the brain, on the other hand, are first responders to the deluge of heme after SAH, and are necessary for aneurysm formation [9,16]. As of yet, no causal relationship has been elucidated between macrophages and DCI. In this study, we show the critical role that microglia play in facilitating vasospasm and neuronal apoptosis. Furthermore, we establish a temporal role for the TLR4 pathway in the induction of apoptosis and vasospasm using both *in vivo* and *in vitro* models.

## Materials and methods

### Materials

Hemin was purchased from Sigma Aldrich (St. Louis, MO, USA) and dissolved in dimethyl sulfoxide (DMSO).

#### Animals

All animal experiments were approved for use by the Beth Israel Deaconess Medical Center Institutional Animal Care and Use Committee and performed in accordance with the National Institutes of Health Guide for the Care and Use of Laboratory Animals. All mice were 10- to 12-week-old males on a C57BL/6 background: TLR4−/−, MyD88−/−, TRIF−/− and wild type (Jackson Laboratory, Bar Harbor, ME, USA).

#### Primary microglial culture

This method is described in detail elsewhere [17]. Briefly, microglia were harvested from neonatal mice (P0-P5) using the Papain Dissociation System (Worthington Biomedical Corporation, Lakewood Township, NJ, USA). The tissue was minced and triturated, then incubated at 37°C for one hour. The suspension was subjected to a discontinuous gradient separation, followed by re-suspension in DMEM-10% FBS containing 1 ng/ml macrophage colony-stimulating factor (M-CSF). The flask was intermittently shaken over the next two to three weeks to obtain a confluent microglial culture.

#### TNF-α ELISA

Primary microglial culture was incubated with 40 μm hemin for 24 hours and TNF-α was measured in supernatant per protocol from BD Biosciences (San Jose, CA, USA).

#### In vitro vasospasm

C57BL/6 mice were anesthetized with isoflurane followed by careful dissection of a 3 cm length of the descending aorta. The aorta was then secured to a vibrotome (Leica Biosystems, Buffalo Grove, IL, USA) plate with glue and 100 μm thick slices were acquired. The aortic slices were incubated in modified Krebs-Henseleit solution containing (mmol/L): NaCl 120, KCl 4.5, MgSO_4_ 1, NaHCO_3_ 27, KH_2_PO_4_ 1, CaCl_2_ 2.5 and dextrose 10. The rings were equilibrated for 90 minutes at 37°C 5% CO_2_ and the medium was replaced every 20 minutes, as described previously [18].

#### In vitro and in vivo vasospasm measurement

Coronal cross sections were dehydrated using alcohol and stained with hematoxylin and eosin for *in vivo* slices. *In vitro* slices of mouse aorta were imaged directly. Images were acquired with Spot Advanced Software (SPOT Imaging Solutions, Sterling Heights, MI, USA). Using measurement tools provided in the software, the inner and outer perimeters were measured and the lumen radius to wall thickness ratio was calculated from these measurements. Three consecutive slices were measured and averaged to obtain the final lumen to wall ratio.

#### SAH

The subarachnoid hemorrhage model was previously described with several modifications detailed below [19]. Mice were anesthetized with xylazine (10 mg/kg) and ketamine (12 mg/kg) and placed in a stereotax where a midline scalp incision was performed. A burr hole was drilled 3.5 mm anterior to the bregma until dural penetration was achieved. A 27-gauge spinal needle was advanced ventrally at 40° to a depth of 5 mm dorsoventral. A total of 60 μl of arterial blood from a donor mouse was injected over 10 seconds.

#### ICV injections

Mice were anesthetized as described above. Two burr holes were drilled 0.22 mm posterior to the bregma, 1 mm lateral, and 2.25 mm in depth to enter the bilateral ventricles. Pulled glass capillaries were used to inject 8 μl of clodronate (a generous gift provided by Prof. Reto Schwendener) or PBS liposomes were divided equally between the ventricles, over 12.5 minutes. The capillaries were held in place for 2.5 minutes thereafter to prevent any regurgitation, followed by skin closure. Intracerebroventricular (ICV) injections were performed on post-operative days (PODs) 1 to 2 after SAH procedure. ICV injections of 5 μg of LPS (lipopolysaccharide) at a concentration of 1 μg/μl were performed at the coordinates noted above in the right lateral ventricle only, as previously described [20]*.* No SAH surgeries were done in these mice.

#### Immunohistochemistry and TUNEL staining

Adult male C57BL/6 were sedated with an Avertin overdose (250 mg/kg), followed by perfusion with ice-cold PBS. The brains were fixed and then cut into 12 μm coronal serial sections with a Leica CM3050 S cryostat. TUNEL staining was performed as per instructions (Roche Diagnostics, Indianapolis, IN, USA). Primary antibodies for Isolectin B1 (Sigma-Aldrich, St. Louis, MO, USA), Glial Fibrillary Acidic Protein (Dako, Carpinteria, CA, USA Toll-like receptor 4 (Santa Cruz Biotechnology, Santa Cruz, CA, USA), or β-III Tubulin (Abcam, Cambridge, MA) were applied at a dilution of 1:250, followed by secondary incubation with Alexa Fluor antibodies at a dilution of 1:250. Fluorescent microscopy was done on a Zeiss Axio Scope (Carl Zeiss, Inc., Thornwood, NY, USA). Brightness and contrast of images were adjusted in Image J software (National Institutes of Health, USA).

#### Statistics

Continuous variables were assessed for normality with skewness and kurtosis. All variables measured in this study were normally distributed and groups were compared with the Student’s *t*-test or ANOVA. If comparisons were made among groups analyzed by ANOVA, the Bonferroni correction was used. All statistical analyses were performed using SPSS 19 software (SPSS Inc., Chicago, IL, USA). P <0.05 was considered statistically significant.

## Results

**Figure 1:**
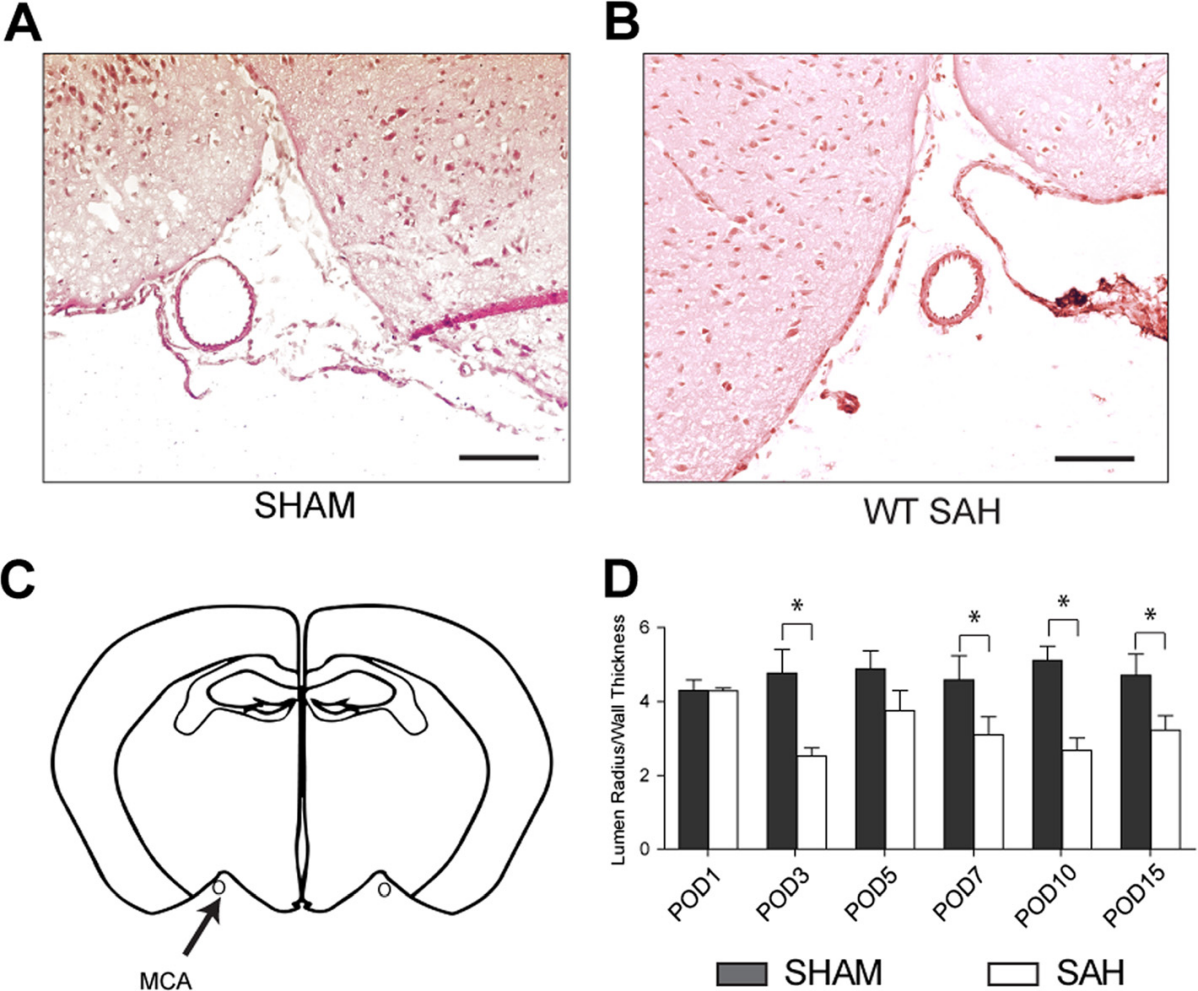
**Vasospasm time course in subarachnoid hemorrhage model. A**. Hematoxylin and Eosin (H&E) stain of coronal section through middle cerebral artery (MCA) in sham from post-operative day (POD) 7. Scale Bars are all: 10 μm. **B**. H&E of MCA stain after subarachnoid hemorrhage (SAH) induction on POD 7. **C**. Shows a diagram of the coronal section where MCA dimensions were measured for vasospasm analysis. **D**. Lumen radius/wall thickness was measured to determine the degree of vasospasm. Student’s *t*-test was done for each POD and significant differences between sham and SAH were noted for POD 3, 7, 10 and 15 with *P*-values of <0.001, <0.04, <0.001 and <0.01, respectively. Error bars are standard deviations and N = 4 per group.

**Figure 2:**
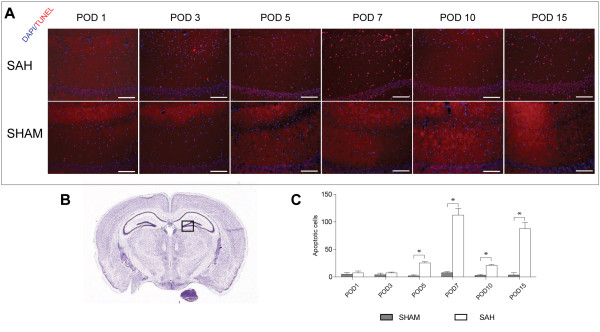
**Neuronal apoptosis time course in subarachnoid hemorrhage model. A**. Merge of DAPI and TUNEL staining in sham and subarachnoid hemorrhage (SAH) along entire time course from post-operative days (PODs) 1 to 15. Scale Bar: 10 μm. **B**. Nissel stain from atlas.brain-map.org showing specific area of dentate gyrus that was used for quantification of neuronal apoptosis. **C**. Apoptotic neurons quantified in dentate gyrus on each POD. Student’s *t-*test for each POD showed significance between sham and SAH on POD 5 (*P* <0.02), 7 (*P* <0.0001), 10 (*P* <0.01) and 15 (*P* <0.0001). Error bars are standard deviations and N = 4 per group.

To determine the optimal time to visualize vasospasm, we performed a time course of subarachnoid hemorrhage from PODs 1 through 15 (Figure 1). The dimensions of the middle cerebral artery were analyzed at the level of the hippocampus for consistency, as shown in Figure 1C. We found that the difference between sham and wild type (WT) SAH vasospasm was maximal on days 3 and 10. There was no difference between sham and WT SAH on POD 5, indicating resolution of vasospasm by Day 5, followed by delayed vasospasm beginning on Day 7 and plateauing on Day 10 (Figure 1). To determine if neuronal cell death correlated with vasospasm in our model, we quantified TUNEL positive cells from days 1 through 15 in the dentate gyrus of the hippocampus. Similarly, cell death also had a bimodal distribution, peaking on PODs 7 and 15 (Figure 2).

**Figure 3:**
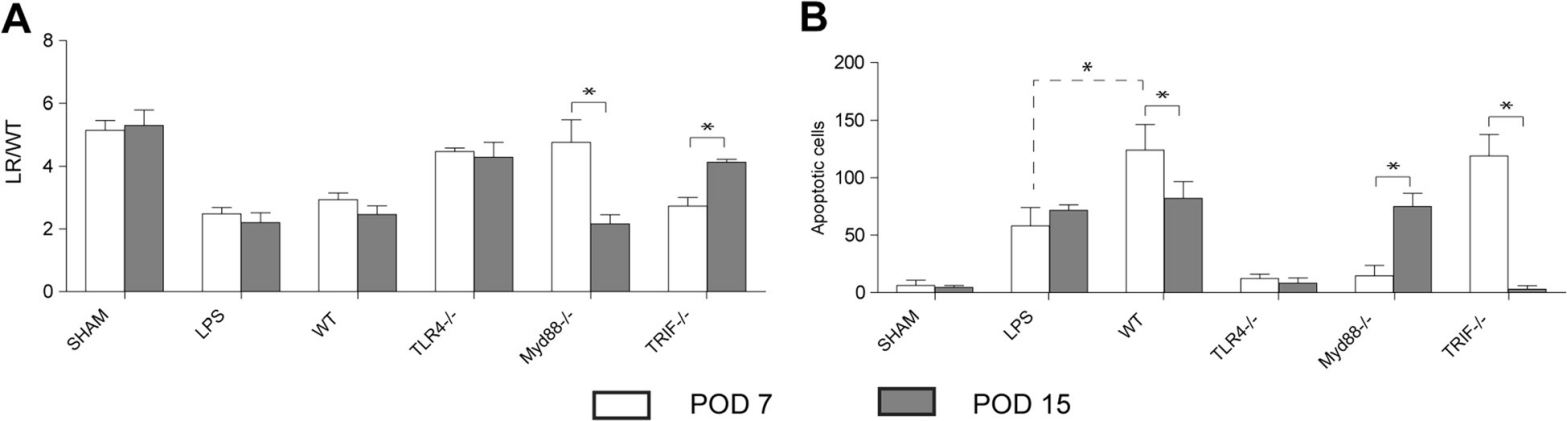
**Quantification of vasospasm and neuronal apoptosis at PODs 7 and 15 in the TLR4 pathway. A**. Vasospasm quantified by lumen radius/wall thickness on post-operative days (PODs) 7 and 15 in sham, intracerebroventricular (ICV)-injected lipopolysaccharide (LPS), wild type subarachnoid hemorrhage (SAH), TLR4−/− SAH, MyD88−/− SAH, and TRIF−/− SAH. Student’s *t-*test showed significantly increased vasospasm on POD 15 in Myd88−/− (*P* <0.01) and TRIF−/− on POD 7 (*P* <0.02). No difference between wild type SAH and TRIF−/− on POD 7 or wild type SAH and MyD88−/− on POD 15. For A and B, error bars are standard deviations and N = 4 for each group. **B**. Apoptotic neurons quantified in dentate gyrus at PODs 7 and 15 in sham, ICV-injected LPS, wild type SAH, Toll-like receptor 4 (TLR4)−/− SAH, MyD88−/− SAH, and TRIF−/− SAH. Student’s *t*-test on POD 7 resulted in significantly increased neuronal apoptosis in TRIF−/− (*P* <0.03) and in MyD88−/− on POD 15 (*P* <0.04). Furthermore, a significant increase in neuronal apoptosis exists between wild type (WT) SAH and ICV LPS on POD 7 by Bonferroni *post-hoc* comparison (*P* <0.04). No difference between wild type SAH and TRIF−/− on POD 7 or wild type SAH and MyD88−/− on POD 15.

We focused on PODs 7 and 15 because neuronal cell death was maximal at these two time points, and it correlated well with human vasospasm peaking on post-bleed Day 7, with continued risk of vasospasm through post-bleed Day 14 [21]. We then set out to determine the role of the TLR4 signaling cascade as it relates to vasospasm and neuronal apoptosis on PODs 7 and 15 (Figure 3). On PODs 7 and 15, vasospasm in the WT SAH was significantly increased compared to the TLR4−/− SAH (Figure 3A). Intracerebroventricular injection of LPS, a known TLR4 agonist, showed similar degrees of vasospasm to the WT SAH on both PODs 7 and 15 [22]*.* Furthermore, when comparing SAH in MyD88−/− to TRIF−/−, vasospasm was significantly greater in the TRIF−/− on POD 7, while the opposite was true at POD 15. Of note, when comparing maximal vasospasm on PODs 7 and 15, there was no difference between WT SAH and TRIF−/− SAH on POD 7, and WT SAH and MyD88−/− SAH on POD 15. Minimal vasospasm was seen in the sham and TLR4−/− SAH on both days.

**Figure 4:**
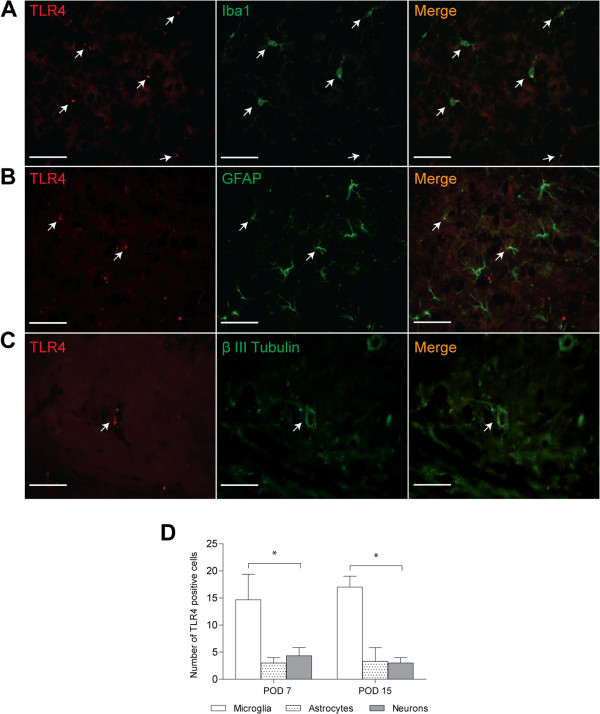
**Immunohistochemistry of TLR4 co-localization among the different cell types of the murine brain.** Representative images from four mice per group and four different fields of view on post-operative day (POD) 7 **A**. Immunohistochemistry across shows staining with Toll-like receptor 4 (TLR4) in the first panel, Iba1 (for microglia) in the second panel, and the merge in the third panel. Arrows delineate co-localization. **B**. Immunohistochemistry showing individual panels and merge for astrocytes with Glial Fibrillary acidic protein. **C**. Immunohistochemistry for β III Tubulin reflecting co-localization of TLR4 and neurons. Scale Bars: 15 μm. **D**. Quantification based on four mice and four different fields of view at POD 7 and POD 15 showing microglia express the most Toll-like receptor 4 (TLR4) by ANOVA at both time points (*P* <0.001).

Additionally, we measured neuronal apoptosis in these groups, as described in Figure 2. Interestingly, TRIF−/− SAH had a statistically equivalent neural apoptotic burden to WT SAH on POD 7, while on POD 15, there was no difference between MyD88−/− SAH and WT SAH. In terms of minimal apoptotic burden, there was no difference between the number of apoptotic neurons quantified in sham, TLR4−/− SAH, and MyD88 −/− SAH on POD 7. At POD 15, there was no difference between the number of apoptotic neurons quantified in sham, TLR4−/− SAH and TRIF−/− SAH (Figure 3B). Of note, the LPS injected mice, demonstrated significantly less neuronal apoptosis at POD 7, compared to WT SAH; however at POD 15 there was no difference between these groups. With the understanding that the TLR4 pathway may play a role in vasospasm and neuronal apoptosis, we wanted to identify what type of cell was expressing TLR4. Microglia, astrocytes and neurons were examined for TLR4 expression with representative images shown in Figure 4A-C and quantification of TLR4 co-localization in Figure 4D. Based on these results, we found that the majority of TLR4 is expressed in microglia at both POD 7 and 15 after SAH.

**Figure 5:**
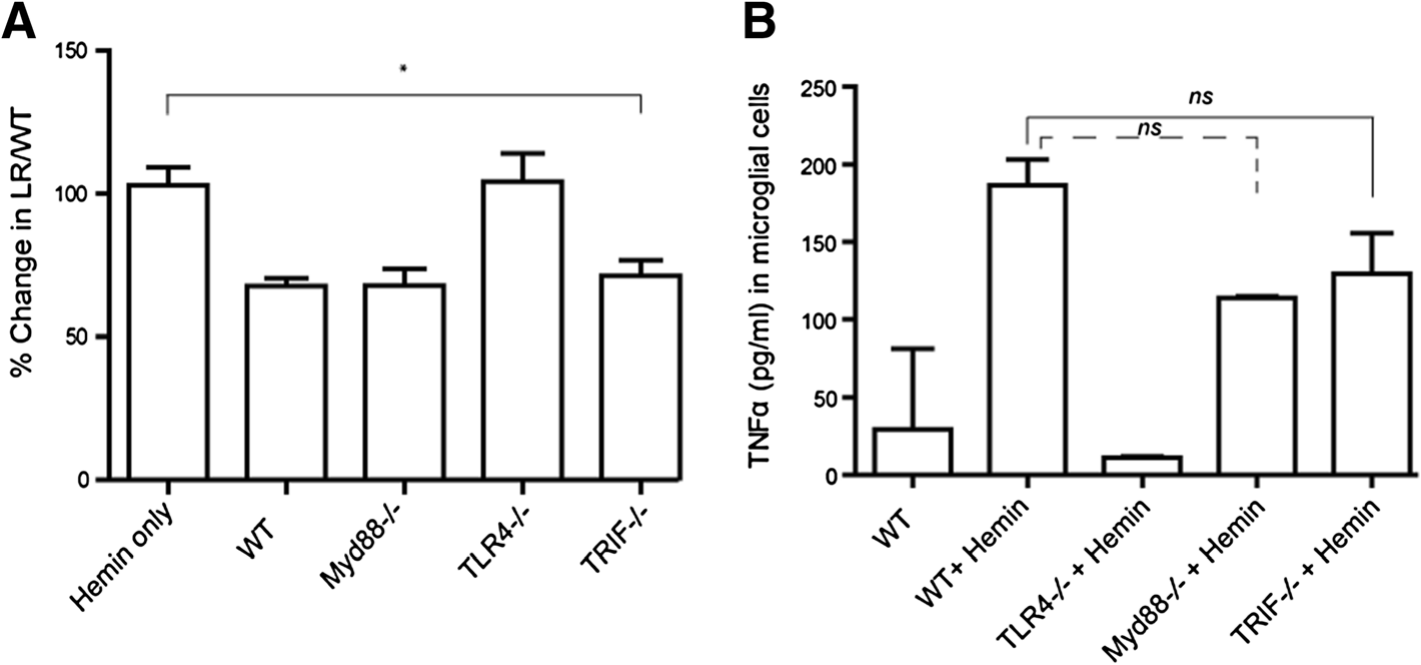
**In vitro vasospasm assay and cytokine production in response to hemin. A**. *In vitro* vasospasm of mouse aortic ring slice after 5-minute incubation with supernatant from primary microglial cell culture (PMG) exposed to 40 μM hemin for 24 hours. Each PMG culture was exposed to one mouse aortic ring and this was repeated four times. Error bars are standard deviations. **B**. TNF-α was measured in the PMG supernatant after 24 hours of hemin exposure. This was repeated four times to get standard deviations in this graph. No difference was seen between wild type (WT), TRIF−/−, and MyD88−/− PMG per the Bonferroni correction. One way ANOVA for both vasospasm and TNF-α secretion were significant (*P* <0.03, *P* <0.02, respectively).

To further elucidate the relationship between microglia and vasospasm, we modified an *in vitro* assay [18] where we incubated neonatal primary microglial (PMG) culture with hemin for 24 hours to simulate the *in vivo* environment after subarachnoid hemorrhage. After 24 hours, the supernatant from the cultures was taken and incubated with axial sections of wild type mouse aortic rings for 5 minutes and vasospasm was measured. PMG from all genotypes, except the TLR4−/− mice, were able to induce vasospasm and secrete TNF-α (Figure 5A, B). Based on these results, the TLR4 receptor is necessary for microglia to secrete some factor into the media that causes vasospasm in mouse aortic slice culture. To exclude endotoxin contamination in our hemin preparation, endotoxin was measured by ELISA and found to be <0.001 ng/μl (data not shown).

**Figure 6:**
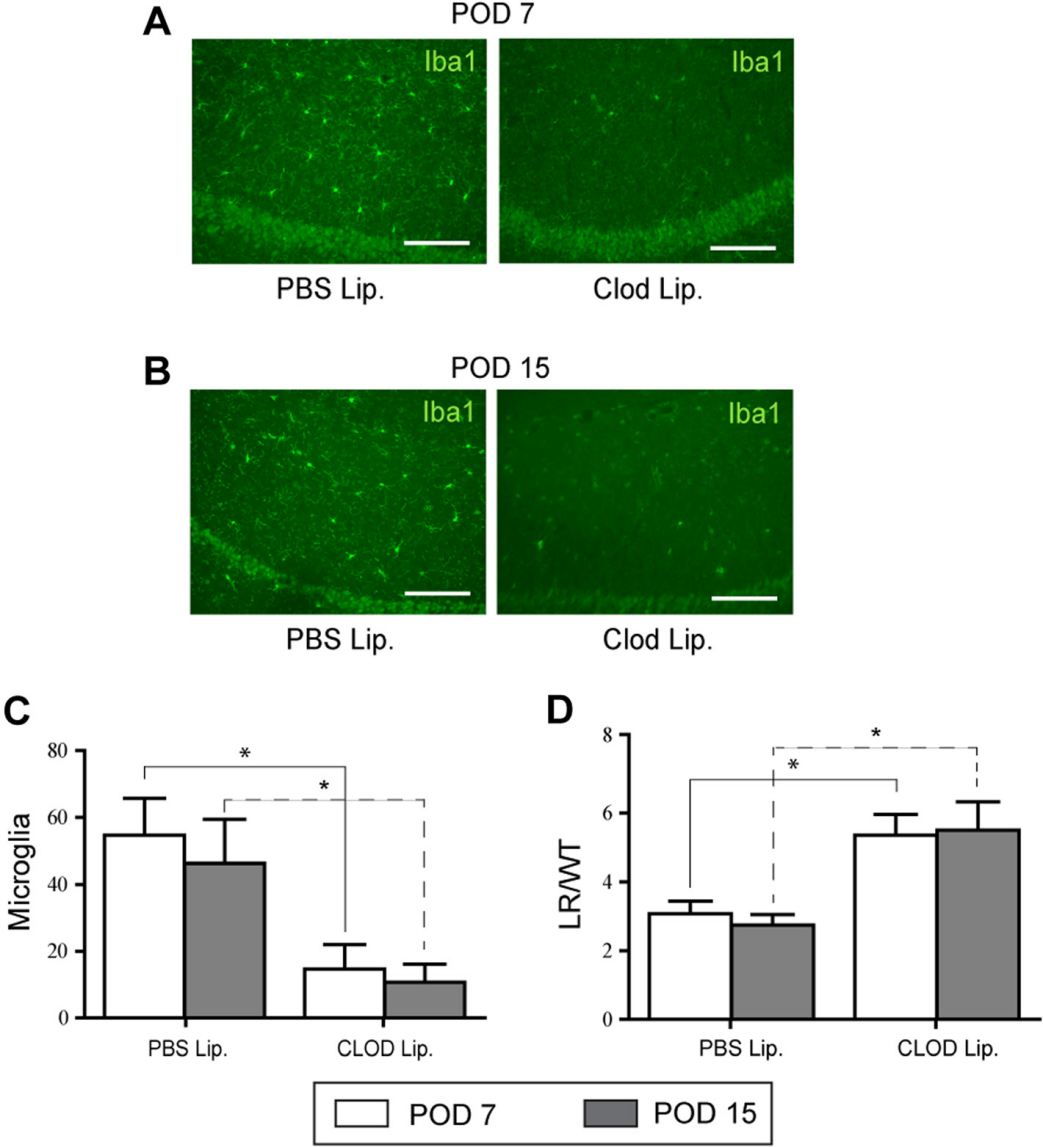
**The effects of microglial ablation on vasospasm. A**. Top panels: microglial staining with Iba1 at the level of the hippocampus of wild type subarachnoid hemorrhage SAH on post-operative days (PODs) 7 and 15 after intracerebroventricular (ICV) injection of control PBS liposomes. **B**. Bottom panels: microglial depletion in wild type SAH on PODs 7 and 15 after ICV injection with clodronate liposomes. Scale Bars: 10 μm in all panels. **C**. Quantification of microglial cells in the hippocampal area of wild type SAH mice with ICV injections of PBS liposomes and clodronate liposomes. N = 3 for each group and error bars are standard deviations. Comparison by Student’s *t*-test on POD 7 and POD 15 between clodronate and PBS liposomes injections shows significant depletion on both days (*P* <0.03). **D**. Measurement of vasospasm shows significant ablation of vasospasm at both time points after microglial depletion by Student’s *t*-test: POD 7 (*P* <0.001) and POD 15 (*P* <0.001). N = 3 mice for each group.

The *in vitro* data suggest that the microglial TLR4 receptor is necessary for vasospasm, as well as the *in vivo* data which indicate that the TLR4 receptor is necessary for vasospasm. To resolve whether microglia are necessary for vasospasm *in vivo*, we depleted microglia *in vivo* via intraventricular injection of clodronate liposomes into WT SAH. Immunohistochemistry for microglia shows virtually complete depletion with clodronate compared to PBS liposomes (Figure 6A-C). Additionally, when vasospasm was measured at PODs 7 and 15, the depletion of microglia with clodronate resulted in significant amelioration of vasospasm at both time points (Figure 6D).

**Figure 7:**
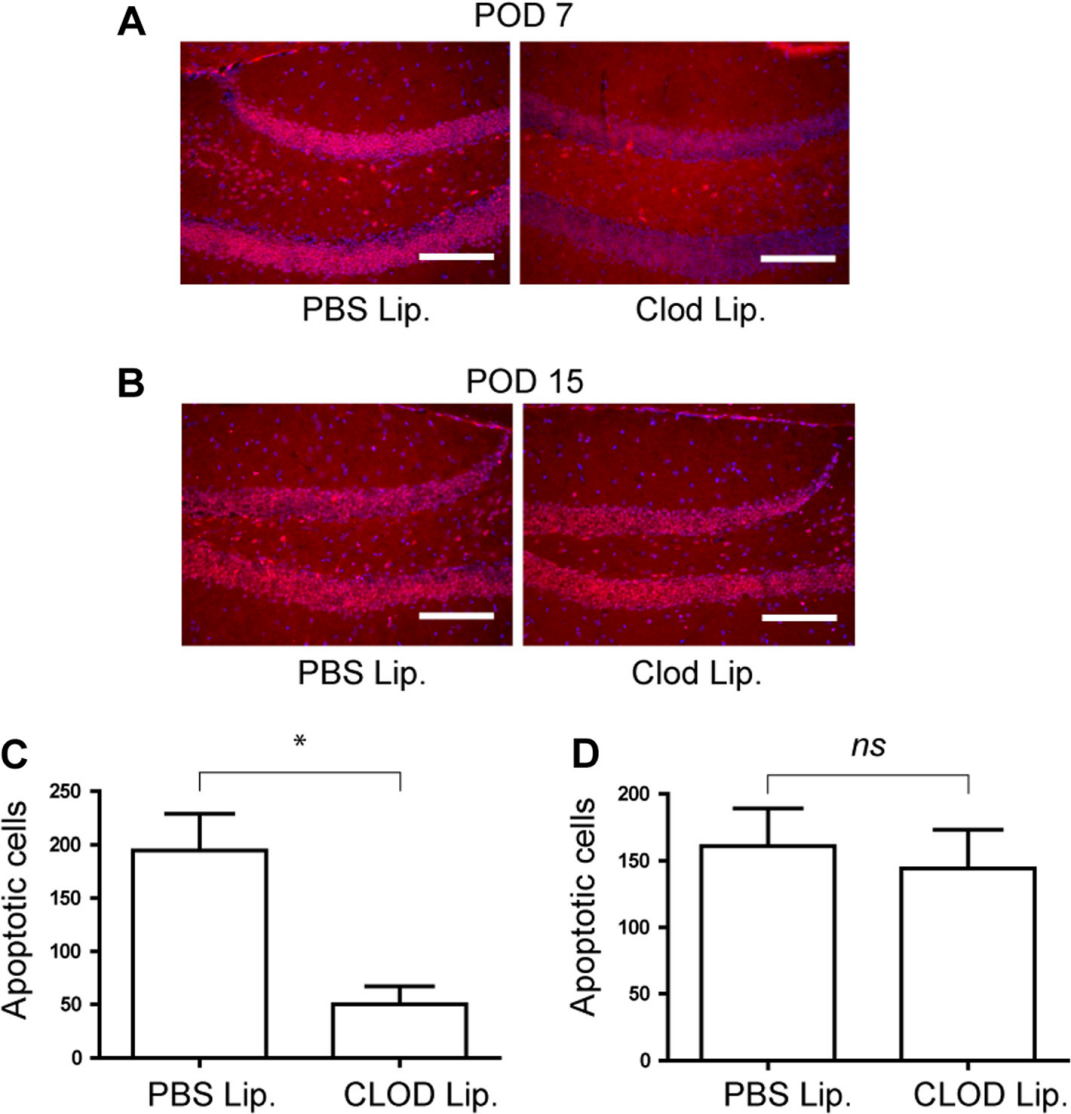
**The effects of microglial ablation on neuronal apoptosis. A**. Top panel: merge of DAPI and TUNEL staining from dentate gyrus of wild type subarachnoid hemorrhage (SAH) after intracerebroventricular (ICV) PBS liposome injection on POD 7 and 15. **B**. Bottom panel: merge of DAPI and TUNEL staining in wild type SAH after ICV clodronate injection on POD 7 and 15. Scale Bars: 10 μm in all panels. **C**. Quantification of apoptotic neurons on POD 7 displaying significant reduction, by Student’s *t*-test (*P* <0.03), after ICV clodronate injection and microglial ablation. **D**. Quantification of apoptotic neurons on POD 15 revealing no change between ICV PBS and clodronate injections. N = 3 for each group and error bars are standard deviations.

Finally, we determined whether microglial depletion effected neuronal apoptosis in Figure 7, and found a similar bimodal theme. At POD 7, the depletion of microglia resulted in a significant decrease in neuronal apoptosis (Figure 7A, C); while at POD 15, microglial depletion had no effect (Figure 7B, D).

## Discussion

In this study, we have elucidated possible roles for microglia and the TLR4 pathway with respect to vasospasm and neuronal apoptosis. Many have suggested a role for TLR4 in SAH and more recently a modulatory role for peroxisome proliferator-activated receptor gamma (PPAR-γ) via the TLR4 pathway [10-12,23]. However, our study is unique in that we attempted to ascertain the roles of different signaling pathways downstream of TLR4 activation and followed the mice out to 15 days to better parallel the human condition where delayed cerebral ischemia can occur up to 21 days from ictus [24].

It is interesting that two phases of vasospasm were observed, one beginning on POD 3, resolving by POD 5, and then another phase beginning on POD 7 and plateauing by Day 10 (Figure 1). This is important to note because it is similar to the human condition in that vasospasm is bimodal, with an early ictal phase followed by a delayed phase; it is the delayed vasospasm that is associated with increased neuronal cell death or delayed cerebral ischemia (DCI) [3,21,25].

Furthermore, our *in vivo* immunohistochemistry data suggest that TLR4 is necessary for neuronal apoptosis. It is interesting to note that neuronal apoptosis seen in WT SAH and TRIF−/− SAH was significantly greater than that seen in LPS on POD 7, despite equivalent degrees of vasospasm (Figure 3). On POD 7, one can infer that some product from red blood cell breakdown is able to induce increased cell-death in the dentate gyrus through the TLR4-MyD88 pathway and downstream signal transduction systems that LPS cannot; however, an insufficient amount of LPS could also be the case*.* Because there was no difference between neuronal apoptosis and vasospasm in WT SAH and TRIF−/− SAH at POD 7, we conclude that some portion of the neuronal damage and vasospasm occurring on POD 7 (early SAH) is TLR4-MyD88-dependent. Likewise, because there was no difference between neuronal apoptosis and vasospasm observed in WT SAH, MyD88−/− SAH and LPS on POD15, neuronal damage and vasospasm on POD 15 (late SAH) are largely TLR4-TRIF-dependent. Based on our results in Figure 3, we see that vasospasm is, for the most part, directly correlated with neuronal apoptosis. Given that vasospasm is either constant or increasing with time, it is expected that the apoptotic burden will not change or decrease only slightly as resolution of the insult occurs. The exception is the TRIF−/− SAH where vasospasm decreases with time and, therefore, we believe that the clearance of the apoptotic burden results in virtually no apoptotic cells by Day 15*.* Furthermore, the temporal relationship between TLR4-MyD88 activation and TLR4-TRIF activation is not unprecedented. Expression of NF-kB is seen in two phases after LPS stimulation of TLR4 [14]. MyD88 is responsible for the early phase and TRIF, the later phase, similar to our results. Because the role for microglia in DCI and SAH is largely unknown with respect to TLR4, we performed immunohistochemistry to verify that microglia do indeed express a majority of TLR4 *in vivo* after SAH at both POD 7 and 15. Although astrocytes and neurons express less TLR4, their role in DCI and cerebral inflammation may still be significant (Figure 4). To verify the relationship between the TLR4 pathway and microglia with respect to vasospasm, we performed *in vitro* vasospasm assays that confirmed a necessary role for the TLR4 receptor (Figure 5). While other groups have implicated microglia and heme in the pathogenesis of intracerebral hemorrhage and shown that TLR4−/− was protective, the downstream mediators of TLR4 were never examined with respect to cytokine production or vasospasm [26].

We found that the TRIF and MyD88 pathways elicited equal degrees of vasospasm, as well as TNF-α secretion, compared to WT microglia. Because vasospasm and TNF-α secretion were not additive in WT microglia stimulated by heme, the temporal theme of sequential activation of MyD88 and TRIF is supported, although not necessarily in that order based on this *in vitro* experiment. A plateau effect is also possible where despite simultaneous activation of the MyD88 and TRIF pathways in WT microglia, the mouse aortic slice cannot constrict further. The other possible explanation is that the determination of the dominant pathway is influenced by the external chemical and cellular milieu of the brain, which is lacking in culture.

To elucidate the role of microglia *in vivo*, with respect to vasospasm and neuronal apoptosis, we depleted microglia using clodronate liposomes and showed that in both early and late phases of SAH, microglia are necessary for vasospasm. Also of note, neuronal apoptosis was abrogated by microglial depletion in early SAH only (Figure 7). With respect to a role for microglia in SAH, to our knowledge, we are the first to effectively deplete this specific cell type in the brain and demonstrate a functional response. Others have noted proliferation and activation of microglia after hemorrhagic stroke, specifically SAH and intracerebral hemorrhage [26,27]. Interestingly, in hemorrhagic stroke, microglia seem to be detrimental by some accounts, whereas in neonatal stroke and neurodegenerative diseases they have a more beneficial role [26,28,29]. The caveat is that effects of microglial depletion were only examined at one time point after depletion. Perhaps, if other time points after microglial depletion had been studied, the role of microglia in the pathogenesis of these diseases would also change with time.

Taken together, our model suggests that there could be different phases of SAH. The early phase of SAH, where neuronal apoptosis is largely TLR4-MyD88-dependent and microglial-dependent, followed by a late phase that is characterized by a TLR4-TRIF dependent, microglial-independent neuronal apoptosis. Furthermore, vasospasm is characterized by an early and late phase response that depends on MyD88 and TRIF, respectively. Finally, microglia seem to be both necessary and sufficient to cause vasospasm in both the early and late phases of SAH, based on our *in vitro* and *in vivo* models (Figures 5 and 6).

These findings may explain why therapies tailored to aSAH patients have failed for the most part. If these data can be translated to the SAH patient population, novel immunotherapies could be conceived that target different components of Toll like receptor signaling and microglia at different points in the patient’s hospital course to alleviate the cerebral inflammatory burden and improve outcomes.

## Abbreviations

aSAH: Aneurysmal subarachnoid hemorrhage; DCI: Delayed cerebral ischemia; DMEM: Dulbecco's modified eagle medium; DMSO: Dimethyl sulfoxide; FBS: Fetal bovine serum; ICV: Intracerebroventricular; IRAK4: IL-1 receptor associated kinase 4; LPS: Lipopolysaccharide; MCA: Middle cerebral artery; MCSF: Macrophage colony-stimulating factor; MyD88: Myeloid differentiation primary response gene; NF-κB: Nuclear factor kappa-light-chain-enhancer of activated B cells; PBS: Phosphate-buffered saline; PMG: Primary microglial; POD: Post-operative day; PPAR-γ: Peroxisome proliferator-activated receptor gamma; SAH: Subarachnoid hemorrhage; TLR4: Toll-like receptor 4; TNF-α: Tumor necrosis factor-alpha; TRIF: Toll receptor associated activator of interferon; TUNEL: Terminal deoxynucleotidyl transferase dUTP nick end labeling; WT: Wild type.

## Competing interests

The author declares that he has no competing interests.
